# Evidence Use in the Development of the Australian Dietary Guidelines: A Qualitative Study

**DOI:** 10.3390/nu13113748

**Published:** 2021-10-23

**Authors:** Kate Wingrove, Mark A. Lawrence, Cherie Russell, Sarah A. McNaughton

**Affiliations:** Institute for Physical Activity and Nutrition (IPAN), School of Exercise and Nutrition Sciences, Deakin University, Geelong 3220, Australia; mark.lawrence@deakin.edu.au (M.A.L.); caru@deakin.edu.au (C.R.); sarah.mcnaughton@deakin.edu.au (S.A.M.)

**Keywords:** dietary guidelines, dietary patterns, evidence synthesis, evidence translation, qualitative research

## Abstract

Dietary guidelines are important nutrition policy reference standards that should be informed by the best available evidence. The types of evidence that are reviewed and the evidence review methods that are used have implications for evidence translation. The aim of this study was to explore perceived advantages, disadvantages, and practicalities associated with the synthesis and translation of evidence from nutrient-based, food-based, and dietary patterns research in dietary guideline development. A qualitative descriptive study was conducted. Twenty-two semi-structured interviews were conducted with people involved in the development of the 2013 Australian Dietary Guidelines (ADGs). Transcripts were analysed thematically. To inform future ADGs, there was support for reviewing evidence on a range of dietary exposures (including dietary patterns, foods and food groups, nutrients and food components, and eating occasions) and health outcomes, as well as evidence on environmental sustainability and equity. At the evidence synthesis stage, practicalities associated with planning the evidence review and conducting original systematic reviews were discussed. At the evidence translation stage, practicalities associated with integrating the evidence and consulting stakeholders were described. To ensure that the best available evidence is translated into future ADGs, evidence review methods should be selected based on the exposures and outcomes of interest.

## 1. Introduction

Dietary guidelines provide recommendations on the foods and dietary patterns that are associated with reduced diet-related chronic disease and obesity risk and provide sufficient amounts of the nutrients required to promote health [1,2,3]. Dietary guideline statements are often accompanied by food guides that are designed to provide practical advice on selecting quantities, combinations, and varieties of foods to achieve healthy dietary patterns [3]. Dietary guidelines can be used to underpin policies and programs across a range of sectors, including health, education, and agriculture [3,4]. They can also be used by health professionals who work directly with members of the public as nutrition education tools [3,4].

The FAO provides guidance to member states on the development of dietary guidelines. The dietary guideline development process typically involves the establishment of an expert committee that is responsible for synthesising and translating evidence into dietary guidelines [1,2,3]. Since the FAO report on the preparation and use of food-based dietary guidelines was published in 1998, perspectives on the types of evidence that should be reviewed and the evidence review methods that should be used have evolved [1,5,6]. The types of evidence that are reviewed and the methods that are used to review the evidence have implications for evidence translation [6,7,8].

According to the FAO, dietary guidelines should be informed by a review of the best available evidence on associations between diet and health [1,2]. Since the emergence of the field of nutrition science in the 1900s, associations between dietary exposures (e.g., nutrients, foods, and dietary patterns) and health outcomes (e.g., micronutrient status, chronic disease risk factors, chronic disease incidence, and mortality) have been explored [9,10]. Dietary patterns are defined by the USDA as the “quantities, proportions, variety, or combination of different foods, drinks, and nutrients in diets and the frequency with which they are habitually consumed” [11] (p. 8). In the last 20 years, the volume of evidence on associations between dietary patterns and health outcomes has increased substantially [12,13]. This evidence is derived primarily from prospective cohort studies that have been conducted over years or decades [14,15,16] examining the associations between dietary patterns and long-term health outcomes, including chronic disease incidence and mortality [17,18,19]. The need to review evidence from dietary patterns research alongside evidence from food-based and nutrient-based research to inform dietary guidelines is now well accepted [7,9,13].

It is now expected that dietary guidelines are underpinned by systematic reviews that are conducted in line with best practice guidelines [5,6,8]. The WHO and Cochrane describe the following steps: define the research question in terms of the Population, Intervention (or exposure), Comparator, and Outcome (PICO) of interest; develop inclusion criteria that reflect the research question; identify studies that meet the inclusion criteria; extract data from included studies; assess the risk of bias associated with each included study; synthesise the data from included studies (using a meta-analysis where possible); assess the quality (or certainty) of the body of evidence for each outcome using the Grading of Recommendations, Assessment, Development and Evaluation (GRADE) system; and interpret the results and draw conclusions [20,21]. In recent years, concerns have been raised that systematic review methods that were developed for other purposes may not be appropriate for use in dietary guideline development [8,22]. For example, the GRADE approach was designed to assess the quality of evidence from randomised controlled trials (RCTs) and non-randomised intervention studies [23]. Depending on the research questions that are asked, approaches that have been designed for the purpose of assessing the quality of evidence from observational studies with complex exposures and long-term health outcomes may be more appropriate [22,24].

At the evidence translation stage, the expert committee considers the quality of the evidence that has been reviewed alongside contextual factors including the social, economic, and political environment [1,2,3]. The dietary guidelines are then drafted, and stakeholders are consulted. As part of the consultation process, dietary guideline statements, food guides, and other resources are tested with consumers (including health professionals and members of the general public) [1,2,3]. The dietary guidelines are then finalised and disseminated [1,2].

The Dietary Guidelines for Australians were first published by the Department of Health in 1982 [25]. Revised guidelines were published in 1992 [26], followed by the publication of specific guidelines for children and adolescents in 1995 [27] and for older adults in 1999 [28]. Revised guidelines were published in 2003 for children and adolescents [29] and for adults [30]. The current Australian Dietary Guidelines (ADGs) were published in 2013 and include five dietary guideline statements and the Australian Guide to Healthy Eating (AGHE) [31,32]. The ADGs were informed by a combination of original systematic reviews and narrative reviews. Our previous analysis demonstrated that most of the systematic reviews on diet and health synthesised evidence from food-based research, while only a small proportion synthesised evidence from dietary patterns research [33]. In July 2020, a review of the ADGs was announced [34]. In developing the next iteration of the ADGs, there is an opportunity to review the latest evidence on associations between dietary patterns and health outcomes. However, the use of systematic review methods that do not take into consideration the nature of dietary patterns evidence may influence evidence translation [7,35]. A description of the challenges associated with conducting the systematic reviews that informed the 2013 ADGs was published in 2014 [36]. A combination of methodological challenges (e.g., accurate assessment of the quality of evidence from prospective cohort studies with dietary exposures) and practical challenges (e.g., the resource-intensive nature of the systematic review process) were described [36]. The aim of this study was to explore perceived advantages, disadvantages, and practicalities associated with the synthesis and translation of evidence from nutrient-based, food-based, and dietary patterns research in dietary guideline development.

## 2. Materials and Methods

A qualitative descriptive study design was used to answer questions about evidence use in dietary guideline development [37,38]. The lead researcher (KW) held a relativist ontological position and used an epistemology that embraced subjectivity [39,40]. This means that she was not looking to identify one ‘absolute truth’ but was instead seeking to explore the participants’ experiences, ideas and opinions. The methods and results of this study have been reported according to the Consolidated criteria for reporting qualitative research (COREQ) checklist [41].

Purposive sampling methods were used to recruit participants [42]. The following people who contributed to the evidence review that informed the ADGs were eligible to participate in this study: members of the 2013 Australian Dietary Guidelines Working Committee (n = 12, excluding ML because his dual roles as a member of the research team and as a member of the Working Committee may be perceived to be a source of potential bias); and members of the Dietitians Association of Australia Review Team (n = 36). The Working Committee was appointed by the National Health and Medical Research Council (NHMRC) and included experts in nutrition and public health and representatives from food industry and consumer groups [31,43]. The Working Committee was responsible for developing the research questions and the evidence review methodology (in line with NHMRC procedures and in collaboration with methodologists), and translating the evidence into dietary guideline recommendations [31]. The Review Team was commissioned by the NHMRC to conduct the evidence review [31,44]. The Review Team included a review leadership team (three senior dietitians), a project officer, a project manager, and 29 reviewers (all dietitians), and two subject librarians [44]. The names and email addresses of eligible participants were identified using publicly available information. A plain language statement and consent form was emailed to eligible participants at the time of recruitment. Informed consent was obtained in writing prior to the commencement of each interview. The concept of information power can be used to determine the sample size for qualitative studies based on the relevance of the information provided by participants [45,46]. Due to the small number of people with the experience required to participate in this study, the sample size was not determined based on the concept of information power [45,46]. Instead, recruitment ceased when no further responses were received from eligible participants. Participants received a $20 WISH eGift Card as compensation for their time.

Semi-structured interviews were conducted online (using Zoom) or over the phone by KW (an Accredited Practising Dietitian and PhD student with training and experience in conducting qualitative research). A semi-structured interview guide was developed and tested in a pilot interview with ML prior to data collection (Table S1) [47]. Participants were asked about their involvement in the dietary guideline development process, the types of evidence that were used to inform the 2013 ADGs, and the types of evidence that should be used to inform future dietary guidelines. The semi-structured nature of the interviews allowed the questions to be tailored to the experiences of each participant [39]. For example, people involved in conducting systematic reviews were asked about the practicalities associated with the evidence synthesis process, and people involved in the development of the dietary guideline statements were asked in more detail about the practicalities associated with evidence translation. Issues relating to the implementation and evaluation of dietary guidelines were beyond the scope of this study.

Throughout the data collection process, minor adaptations to the interview guide were made based on responses from interviewees (e.g., broad questions that were poorly understood were clarified, or additional prompts were added) and in response to the release of new information from the NHMRC about the ADG review process. To enhance reflexivity, KW completed a written reflection on the interview process immediately after each interview [39,42]. The reflection template included factors that may have influenced the data (e.g., variation in interview technique) and preliminary data analysis ideas. Interviews were audio-recorded and transcribed verbatim using artificial intelligence software (Otter.ai). Transcripts were checked for accuracy and edited accordingly by KW, and any information that could be used to identify a participant was removed [48]. Transcripts were not returned to participants.

An iterative thematic approach to data analysis was used, which is consistent with the qualitative descriptive research design [37]. An inductive, open coding technique was used, whereby previously undefined codes were assigned to pieces of data [39,49]. A 10% sample of interview transcripts (n = 3) was independently analysed by two researchers (KW and CR). Each researcher developed a preliminary coding framework to facilitate identification of themes in the data, and the differences between these coding frameworks were discussed [49,50]. The remaining interviews were analysed by one researcher (KW). The coding framework was adapted throughout the data analysis process until the themes provided an accurate representation of the data [37]. NVivo software (Version 12 Plus) was used to facilitate data analysis [51].

## 3. Results

**Figure 1:**
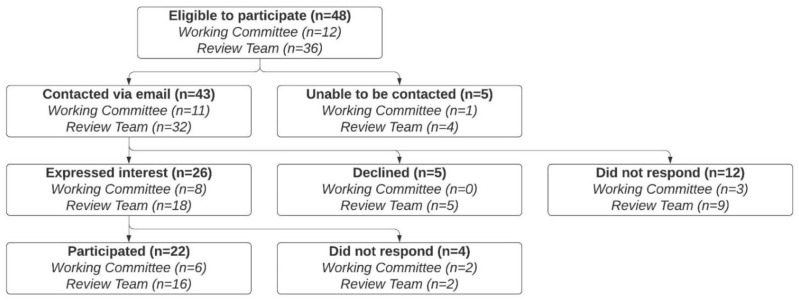
Recruitment and participant characteristics.

Forty-eight people were eligible to participate in this study (Figure 1). Five people were unable to be contacted via email (email addresses were identified, but email delivery failed). Forty-three people were contacted. Twelve people did not respond, three people declined due to a lack of time, and two people declined due to a perceived lack of expertise. Twenty-six people expressed interest in participating; however, four of these people did not respond to follow-up emails. Individual semi-structured interviews were conducted with the remaining 22 people between October and December 2020. Six participants were members of the Working Committee, and 16 participants were members of the Review Team. The length of the interviews ranged from 30 to 60 min.

Eight themes emerged from the data and were organised under three categories (Table 1). Four themes related to exposures and outcomes of interest, two themes related to practicalities associated with evidence synthesis, and two themes related to practicalities associated with evidence translation. Some participants also shared their views on the broader contextual issues associated with dietary guideline development in Australia (such as the processes used to establish the Working Committee and the need for regular, planned revisions of the dietary guidelines). Although these are important issues, they were outside the scope of the aim of this study and, therefore, have not been described in further detail.

### 3.1. Exposures and Outcomes of Interest

In relation to the evidence review that will be conducted to inform the next iteration of the ADGs, participants identified a range of dietary exposures and health outcomes of interest. The importance of reviewing the latest evidence on environmental sustainability and equity was also described. Opinions on the research questions that should be prioritised reflected participants’ views on the public health nutrition issues that future dietary guidelines should aim to address, and their perspectives on the current state of knowledge on relationships between particular exposures and outcomes.

#### 3.1.1. Dietary Exposures

The following dietary exposures of interest were identified: dietary patterns, foods and food groups, nutrients and food components, and eating occasions. Conceptually, dietary patterns, foods and food groups, and nutrients and food components are exposures that reflect dietary intake (i.e., ‘what’ people eat), whereas eating occasions are exposures that reflect eating behaviours (i.e., ‘how’ people eat) [52]. Dietary patterns and foods and food groups were consistently described as important exposures. Some participants described particular nutrients and food components as important exposures in relation to particular health outcomes. Views on the importance of reviewing evidence on eating occasions were mixed.

In line with the food synergy theory [16,18], dietary patterns were described as exposures of interest on the basis that it is the combinations of foods that appear to be most influential on important health outcomes. For example:

“… if we’re defining health in terms of maximising functionality and preventing chronic disease, which is a public health goal, then we need to accept that it’s not a single food, it’s not a single nutrient, it’s actually a package, which we are now calling dietary patterns, which influences those outcomes, and it doesn’t happen quickly.”

[Participant 3, Working Committee].

Food and food groups were described as exposures of interest because although people consume dietary patterns, not every dietary pattern includes every food. For example:

“… I guess if you’ve got a dietary pattern, it might not include all the different foods. Whereas if you’ve got food studies, for example, we know that fatty fish is high in omega-3. Those studies are still important, because that’s a strong source of that particular nutrient”

[Participant 20, Review Team].

Some participants identified particular food groups of interest (e.g., meat, dairy, discretionary foods), explaining that new evidence has emerged since the current ADGs were developed.

Nutrients and food components were described as exposures of interest on the basis that we need to understand the biological mechanisms that underpin associations between particular foods and health outcomes. For example:

“… since we’re talking about the biological aspects, we do need to have a good understanding of the molecular basis for how food components influence health, and that’s actually the nutrient side of it. But it’s not just nutrients, it’s the things we don’t call nutrients, like phytocomponents, and it’s also the interaction between nutrients that occur within the food delivery system”

[Participant 3, Working Committee].

The following nutrients and food components were described as exposures of interest in relation to particular health outcomes: different types of saturated fat in relation to blood lipids, different types of carbohydrate (e.g., sugars compared to starches) in relation to body weight, potassium (including the potassium to sodium ratio) in relation to cardiovascular disease risk, flavonoids in relation to vascular health, and food additives (including stabilisers) in relation to inflammation.

Eating occasions (including the timing and frequency of meals, as well as contextual factors such as eating with others, eating away from home, and eating home-prepared meals) were described as exposures of interest by some participants on the basis that there is emerging evidence to suggest that these exposures may be associated with a range of important health outcomes. Other participants were of the opinion that research questions about ‘what’ people should eat should take priority over research questions about ‘how’ people should eat.

#### 3.1.2. Health Outcomes

Cardiovascular disease, type 2 diabetes, cancer, and overweight and obesity were often described as important health outcomes. The following health outcomes of interest were also identified: gut health (including the microbiome), gastrointestinal diseases, food allergies, mental health, immune function, vitamin D deficiency, healthy ageing, sarcopenia, frailty, osteoarthritis, and cognitive outcomes (including dementia and Alzheimer’s disease).

#### 3.1.3. Environmental Sustainability

There was strong support for an increased focus on environmental sustainability in the development of future dietary guidelines on the basis that it is not possible to promote public health without considering sustainability and in response to increasing environmental threats to food security in Australia. For example:

“So the first thing I’d say is that you don’t even have to put those two things together. You don’t even have to say health and environmental sustainability, because anything that degrades environmental sustainability eventually degrades human health, and that’s becoming more and more accepted”

[Participant 12, Review Team].

Some participants commented on the broad nature of the term ‘environmental sustainability’ and explained that there are many research questions that could be asked. Reviewing the latest Australian evidence on the environmental impacts associated with food production and consumption was identified as a priority due to an increase in the volume of relevant evidence. For example:

“We now have much more evidence than we had back then. And not just of modelling studies of actual observational data. So in terms of environment and food related health, so we’ve got much more data”

[Participant 4, Working Committee].

Other participants explained that although environmental sustainability was important to consider, the evidence review should focus on dietary exposures and health outcomes, and environmental sustainability should be considered as part of the evidence translation process.

#### 3.1.4. Equity

There was strong support for an increased focus on equity in the development of future dietary guidelines on the basis that access to food is a social determinant of health and in response to increasing economic threats to food security in Australia. Some participants explained that ‘equity’ is a broad term, and that there are many research questions that could be asked. Reviewing the latest Australian evidence on relationships between socioeconomic status and dietary intake was identified as a priority. For example:

“So if there’s anything I think we should be looking at, it’s the relationship between socioeconomic status and dietary intake. I’m not a food security expert by any means, I’ve just been an interested observer during the pandemic, and how people’s food skills, so utilisation has been poor, worse than that, we’ve got people economically doing it tough, not having enough to eat. So I think if there’s any time to be asking questions, that’s an important one to be asking of the literature”

[Participant 22, Review Team].

Other participants explained that although equity issues were important to consider, the evidence review should focus on dietary exposures and health outcomes, and equity should be considered as part of the evidence translation process.

### 3.2. Practicalities Associated with Evidence Synthesis

In relation to the evidence synthesis stage of dietary guideline development, practicalities associated with planning the evidence review and conducting original systematic reviews were described.

#### 3.2.1. Planning the Evidence Review

The importance of having an appropriate methodology in place before the evidence review begins was emphasised by some participants. For example:

“So rather than say, ‘go and do some dietary guidelines again’, it’s ‘we thought about how this needs to be done, now here’s the methodology’. And then it’s like the rules of the game have been stipulated before everyone goes on the field; it’s much more functional”

[Participant 3, Working Committee].

Many participants described the importance of balancing evidence review methods with available resources, including human resources, financial resources, and time. The advantages and disadvantages of conducting original systematic reviews for the purpose of dietary guideline development (rather than using existing systematic reviews that were conducted for other purposes) were discussed. The resource-intensive nature of conducting original systematic reviews was emphasised, and there was support for using a combination of original and existing systematic reviews on this basis. If existing systematic reviews are used, the importance of assessing the quality of those reviews and assessing the applicability of the evidence to the Australian context was described. On this basis, some participants argued that the process of identifying and assessing existing systematic reviews can be less efficient than conducting original systematic reviews.

#### 3.2.2. Conducting Original Systematic Reviews

In relation to conducting original systematic reviews, practicalities at the following stages of the systematic review process were described: defining the research question, developing the search strategy and inclusion criteria, assessing the risk of bias associated with individual studies, and assessing the quality of the body of evidence. The importance of managing conflicts of interests and engaging people with expertise in nutrition throughout the systematic review process was also described.

To maximise efficiency, the importance of having clearly defined research questions from the outset was often described. Having clear definitions of the exposures of interest was considered particularly important (e.g., ‘intake of foods high in sodium’ compared to ‘sodium intake’ and ‘fresh meat’ compared to ‘processed meat’). For example:

“… I remember specific points of discussion around, how is [meat] dealt with? You know, what’s red meat? What’s white meat? What’s processed meat? What’s not?”

[Participant 17, Review Team].

Some participants described the importance of tailoring the search strategy and the inclusion criteria to the research question so that relevant evidence is not missed or excluded. For example, the inclusion criteria for the date of publication may vary because the evidence that is most suitable for answering a particular research question may have been published during a particular time period. The inclusion criteria for study design may also vary because some study designs are more suitable for addressing particular research questions than others. For example, in relation to evidence on environmental sustainability:

“So we have to be prepared to stand back and look at the way in which evidence is constructed in different disciplines, because what you don’t want is to say we’re going to have to have a whole lot of systematic reviews [on environmental sustainability], find there’s two studies, and then find we can’t say anything, because there isn’t any research, but there’s probably a lot of research, it’s just not constructed in that way”

[Participant 3, Working Committee].

The importance of selecting risk of bias assessment tools that are appropriate for particular research questions and particular study designs was emphasised. Some participants suggested that risk of bias tools should consider unique factors associated with conducting studies with dietary exposures, including the accurate estimation of dietary intake, and difficulties associated with blinding. For example:

“I think it’s always useful to have tools that are appropriate for the study designs and the questions being asked. Most of the risk of bias, certainly the Cochrane Risk of Bias tools, downgrade many nutrition studies, because of the problems with blinding, [but] blinding is quite difficult, so that needs to be managed in some way”

[Participant 15, Review Team].

The importance of using the most appropriate method for assessing the quality of the body of evidence was emphasised. There were mixed views on the suitability of the GRADE approach. For example, some participants explained that GRADE allows evidence from observational studies to be upgraded when particular criteria are met, which was described as an advantage. Others argued that alternative approaches may be more suitable for assessing the quality of evidence derived from studies with dietary exposures. For example:

“I think on reflection, it would be good to have a system which is specific to dietary type studies, because you’re hardly ever going to get RCTs in this kind of field. They’re more likely to be cohort studies or large population studies. And that doesn’t make them bad. But when you rate them in traditional systems, they always look like low quality evidence. So I think whatever system that is used going forward, there needs to be a process for rating the evidence provided by those kinds of studies more appropriately”

[Participant 20, Review Team].

Some participants described the importance of having a clear process in place to assess the applicability of evidence. For example, it was noted that most dietary patterns evidence is derived from studies conducted in other countries, which may not be applicable to the Australian context:

“… my assumption would be that a lot of the [dietary pattern] studies would not be necessarily conducted in Australia, but they may be European or US studies that could be applied to our Australian context. Again, same thing in reverse, there may be some that may not be relevant at all. And we need to have a process in place where we start to work out, how do we deem them as relevant and what would then be appropriate to be considered for the Australian context”

[Participant 9, Review Team].

Practicalities associated with the management of actual, potential, and perceived conflicts of interest throughout the systematic review process were discussed. Some participants described the importance of considering conflicts of interest within the evidence base. For example:

“I think something that we’ve recognised is that the scientific literature has conflicts of interest within it. And we did include sources of funding. When we did the literature review, we extracted data on the sources of funding. But I think that this time, we would pay more careful attention to that, and perhaps highlight that”

[Participant 21, Review Team].

The importance of managing conflicts of interest within the systematic review team was also highlighted. For example:

“So I think it’s really important in the future that there’s a lot of oversight to make sure that we don’t have reviewers who have vested interests, [or] funding for other work”

[Participant 2, Working Committee].

Some participants described the importance of engaging people with expertise in nutrition throughout the systematic review process on the basis that people without nutrition expertise may misinterpret the evidence. For example:

“I think you do need people who understand nutrition to do it. There’s lots of professional, systematic reviewing people, but honestly, if they don’t understand nutrition, it can lead to erroneous conclusions”

[Participant 21, Review Team].

### 3.3. Practicalities Associated with Evidence Translation

In relation to the evidence translation stage of dietary guideline development, practicalities associated with evidence integration and stakeholder consultation were described.

#### 3.3.1. Integrating the Evidence

Integrating evidence from multiple systematic reviews with different exposures and outcomes, making decisions about the quality of evidence that is required to inform recommendations, and balancing the potential health, economic, social, and environmental consequences associated with particular recommendations were described as important processes that require professional judgement. For example:

“So theoretically, we might be getting reasonably close to saying, these are the combinations of foods that we think will give the best outcomes in terms of population health. But then you have to ask yourself, if the population is to consume this way, how good are we at providing it? And what is the impact on the environment of us producing it? What is the economic cost? Is this something that we trade in? How important is it from the point of view of the country’s GDP? And then the social side of it, which groups in our society actually eat like this? And what are the consequences of us saying they have to eat this other way?”

[Participant 3, Working Committee].

Views on whether dietary guideline statements should focus on dietary patterns or on foods were mixed. Some participants supported the development of dietary guideline statements that describe healthy dietary patterns in broad terms (e.g., consuming a diet that is predominantly plant-based but includes small quantities of minimally-processed animal products). Others explained that dietary guideline statements should continue to focus on foods because that is what members of the general public are likely to understand.

Some participants explained that healthy dietary patterns may include small amounts of unhealthy foods, so, by focusing on dietary patterns, there is a risk that messages about unhealthy foods can get lost or used to the advantage of food industry stakeholders. For example:

“I guess one challenge with the dietary patterns approach is that any unhealthy food can be part of a healthy diet pattern, as far as our food industry friends would be concerned. So that’s a bit of a downside, in that we play into this rhetoric of, ‘it’s all about the total diet’ when really we do want to be highlighting some foods, all the discretionary foods basically, and having clear messages about those. I think that’s where it gets a bit difficult”

[Participant 10, Working Committee].

To address some of these challenges, it was suggested that dietary guidelines should continue to focus on foods but also incorporate clear messages about variety, balance, and moderation.

Views on the food classification system that should underpin the dietary guidelines were mixed. Some participants were of the opinion that the current food classification system that includes five core food groups and ‘discretionary foods’ is not well understood by members of the general public. Other participants highlighted the value of consistent messaging and were concerned that the introduction of a new food classification system could lead to confusion about which foods are ‘healthy’ or ‘unhealthy’. For example:

“…I do wonder if as nutrition scientists, we actually should just get to the point where we call out what’s not a very healthy food and should be consumed in small amounts. And we all agree on that. If we just keep inventing classification after classification, and spending all our time on that, then it actually may play into the hands of interests that are not so interested in people having a healthy diet”

[Participant 21, Review Team].

Some participants supported the development of an Australian-specific version of the NOVA food classification system that classifies foods according to level of processing. Others did not support the use of the NOVA system because they were concerned that consumers may not understand the differences between the four food groups (minimally processed foods, processed culinary ingredients, processed foods, and ultra-processed foods) and, instead, assume that any kind of food processing makes a food ‘unhealthy’.

There was limited support for the development of recommendations about eating occasions due to the following risks: statements that promote eating with others might have negative implications for people who live alone; statements that encourage home-prepared meals might have negative implications for people who do not have the capacity to prepare meals at home; concerns that home-prepared meals are not necessarily healthy and that meals eaten away from home are not necessarily unhealthy were expressed; statements about *how* people should eat might take the focus away from statements about *what* people should eat; and statements that focus on eating occasions could be used to the advantage of food industry stakeholders. For example:

“… the more that we sort of broaden it out with other messages, the more the food industry can say ‘Well, it’s all about enjoyment. We have a dietary guideline about enjoying meals with families and family and friends. And here is our McDonald’s meal on a Friday’…”

[Participant 10, Working Committee].

Some participants expressed support for recommendations that focus on health promotion rather than disease prevention and messages that focus on short-term rather than long-term benefits on the basis that these messages may be more appealing to members of the general public. For example:

“I think we all have a bit of a short-term view of things, and we all feel a little bit invincible. So making it closer in terms of timeframes. So feeling well, not getting sick, being able to do the things that you want to do, so being able to enjoy life, particularly as people get older”

[Participant 15, Review Team].

The importance of considering the environmental consequences associated with the production and consumption of the foods and dietary patterns that are recommended was emphasised. However, to avoid unintended health consequences, it was also noted that recommendations that encourage lower consumption of meat and dairy (for environmental reasons) should be balanced by recommendations that ensure that nutrient reference values for iron and calcium can be met.

From an equity perspective, the importance of ensuring that the foods and dietary patterns that are recommended are commonly available, accessible, and able to be utilised was emphasised. For example:

“So there’s two layers of evidence, first of all, which foods and which compounds in foods and which combinations of foods are best for health. And there could be a number of different ways you could eat, we know that. But then the second layer is, which of these combinations are achievable in terms of our cuisine, in terms of people’s food preferences, in terms of accessibility and sustainability for ensuring equitable food access?”

[Participant 16, Review Team].

Some participants described the importance of involving people with expertise in nutrition in the drafting of dietary guideline statements and the development of food guides. For example:

“… the food guide is essential in taking all of those technical reviews and all of the actual guidelines and translating that for the public. So if people with all of that skill and expertise are not involved in that really important next step, it sort of negates the process. Because if you can’t get the message across properly, then there’s no point in having dietary guidelines...”

[Participant 16, Review Team].

#### 3.3.2. Consulting Stakeholders

To inform the wording of dietary guideline statements and the development of food guides and other resources, the need for a comprehensive and well-resourced consultation process was often described. For example:

“… we always think that we know best, and we actually don’t know best, we don’t know what the consumer and what the person out there on the street, what message they receive. And we need to just be investing much more time in trying to understand that. But we’re not, we’re going to go through the whole process of doing a million [systematic reviews], synthesising the evidence, which is all good and well, but then we fail at the most important step, which is understanding how consumers understand”

[Participant 16, Review Team].

The importance of engaging people from a diverse range of socioeconomic and cultural backgrounds in the consultation process and managing the influence of food industry stakeholders with conflicts of interest was often described. Some participants explained that a robust evidence review process can reduce the influence that submissions from stakeholders with conflicts of interest have on the final version of the dietary guidelines. For example:

“When you can say, look, we’re basing it on proper systematic reviews. And that was the good thing about the last one […] we could say, well, this was the evidence that we were using and this was why…”

[Participant 1, Review Team].

## 4. Discussion

The aim of this study was to explore perceived advantages, disadvantages, and practicalities associated with the synthesis and translation of evidence from nutrient-based, food-based, and dietary patterns research in dietary guideline development. To inform the next iteration of the ADGs, there was support for reviewing evidence on a range of dietary exposures (including dietary patterns, foods and food groups, nutrients and food components, and eating occasions) and health outcomes. In response to increasing environmental and economic threats to food security in Australia, the importance of reviewing the latest evidence on environmental sustainability and equity was described. At the evidence synthesis stage, practicalities associated with planning the evidence review and conducting original systematic reviews were discussed. At the evidence translation stage, practicalities associated with integrating the evidence and consulting stakeholders were described.

The expert committee is typically responsible for identifying and prioritizing the research questions that will be addressed in the evidence review [1,2]. In this study, participants consistently described dietary patterns as important exposures, whereas views on the advantages of reviewing evidence on eating occasions were mixed. Reviews of evidence on dietary patterns and eating occasions have been conducted to inform dietary guidelines in other countries. For example, a series of systematic reviews on dietary patterns and body weight, cardiovascular disease, and type 2 diabetes was conducted to inform the 2015 Dietary Guidelines for Americans [53]. To inform the 2020 Dietary Guidelines for Americans, evidence on the associations between eating frequency and health outcomes and eating frequency and diet quality was also reviewed [11]. The current Dietary Guidelines for the Brazilian Population were informed by a review of evidence on modes of eating (such as eating at regular times and eating with other people) as determinants of healthy diets [54]. A combination of existing and original systematic reviews of relevant evidence on dietary patterns and eating occasions (as exposures) and a range of health outcomes could be used to inform future ADGs.

Research questions about dietary exposures and long-term health outcomes (e.g., chronic disease incidence) can be difficult to address using RCTs due to challenges associated with compliance to dietary interventions and the costs associated with delivering interventions over long periods of time [7,16,55]. Prospective cohort studies are more suitable for addressing these types of research questions but are subject to risk of bias associated with confounding [14,55]. To ensure that the quality of relevant evidence from observational studies is assessed accurately, study participants described the importance of using appropriate risk of bias tools, including tools that consider unique challenges associated with conducting observational studies with dietary exposures (e.g., accurate estimation of dietary intake and difficulties associated with blinding). In Australia, the latest NHMRC Guidelines for Guidelines handbook highlights the importance of using an appropriate risk of bias assessment tool, noting that, “depending on the type of research question, strong observational studies can at times provide more reliable evidence than flawed randomised trials” [56]. Cochrane risk of bias tools exist for randomised trials (ROB2) [57] and non-randomised studies of interventions (ROBINS-I) [58]. These tools consider the sources of bias associated with particular study designs, but were not developed specifically for assessing studies with dietary exposures. In contrast, the Risk of Bias for Nutrition Observational Studies (RoB-NObs) Tool was created by the USDA’s Nutrition Evidence Systematic Review team and used in dietary guideline development [11]. In the development of future ADGs, the use of tools that have been developed to assess the risk of bias associated with observational studies focused on dietary exposures should be considered.

The importance of using an appropriate tool to assess the quality of evidence from observational studies with dietary exposures was described by study participants. Using the GRADE approach, evidence from RCTs is rated more highly than evidence from non-randomised studies from the outset, regardless of the research question [8,23]. The NHRMC guidelines recommend the use of GRADE for assessing the certainty of the evidence but acknowledge the ongoing debate about whether GRADE is the most appropriate system to use in public health guideline development [59]. This debate is also evident in nutrition science literature. For example, Zeraatkar et al. recommend the use of GRADE in dietary guideline development on the basis that this system is the most rigorous [6]. Conversely, Tobias et al. acknowledge the strengths of GRADE but argue that alternative approaches that may be more appropriate for assessing the certainty of evidence from observational studies with dietary exposures should also be considered [22]. In the development of future ADGs, the use of alternative evidence assessment approaches such as the Hierarchies of Evidence Applied to Lifestyle Medicine (HEALM) approach could be considered for research questions that cannot be addressed using RCTs [24].

At the evidence translation stage, integrating the evidence, making decisions about the quality of evidence that is required to inform recommendations, and balancing the potential consequences of recommendations were described as processes that require professional judgement. Blake et al. analysed dietary guideline development processes in 32 countries [5]. They found that in 28 countries, the dietary guidelines committee used an unstructured consensus process to translate the evidence into recommendations. In the remaining four countries, information on the evidence translation process was not available [5]. The need for increased transparency at the evidence translation stage of dietary guideline development has been recognised [60,61]. A number of frameworks now exist to guide decision making in the translation of evidence into recommendations, including the GRADE Evidence to Decision Framework and the WHO-INTEGRATE framework [62,63]. Each of these frameworks includes criteria on the quality of evidence and balancing health benefits and harms. The WHO-INTEGRATE framework also incorporates social, economic, and environmental dimensions [63]. In the development of future ADGs, the use of a suitable framework to guide the translation of evidence into recommendations should be considered.

**Figure 2:**
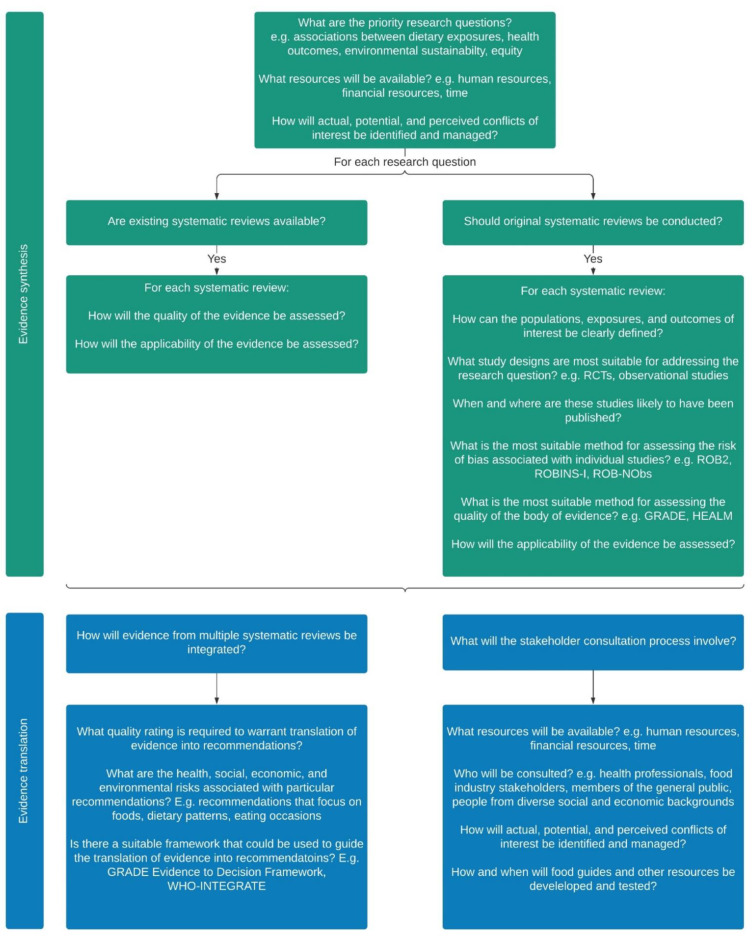
An overview of the practicalities associated with evidence synthesis and translation in dietary guideline development.

Figure 2 provides an overview of the practicalities associated with evidence synthesis and translation in dietary guideline development, summarising the results of this study in the context of approaches that have been described as best practice in the literature.

By exploring the ideas and opinions of people with first-hand experience, this study provides insights into the dietary guideline development process in Australia. Although the sample size was not based on the concept of information power, it is likely that a high level of information power was reached because interviews were conducted with a purposive sample of participants, including 6 out of the 12 eligible members of the Working Committee and 16 out of the 36 eligible members of the Review Team, and the quality of the dialogue was strong [45]. However, it is possible that certain information was not captured because not everyone who was eligible to participate was interviewed. To increase rigor in the qualitative approach, strategies to enhance reflexivity were used, and a subset of transcripts were analysed by multiple researchers [39,42]. A limitation of this study is that dietary guidelines are designed to be context-specific, which limits the transferability of results [42]. However, the practicalities associated with evidence synthesis and translation that were identified may be relevant in other contexts.

## 5. Conclusions

Dietary guidelines are important nutrition policy reference standards that should be informed by the best available evidence. This study explored perceived advantages, disadvantages, and practicalities associated with the synthesis and translation of evidence in dietary guideline development. To inform future ADGs, there was support for reviewing evidence on the associations between a range of dietary exposures (including dietary patterns, foods and food groups, nutrients and food components, and eating occasions) and health outcomes along with the latest Australian evidence on environmental sustainability and equity. To ensure that the best available evidence is translated into dietary guidelines, the most appropriate evidence review methods should be selected based on the exposures and outcomes of interest.

## Figures and Tables

**Table 1:** Overview of the eight themes that emerged from the data, organised under three categories.

Categories	Themes
Exposures and outcomes of interest	Dietary exposures Health outcomes Environmental sustainability Equity
Practicalities associated with evidence synthesis	5. Planning the evidence review 6. Conducting original systematic reviews
Practicalities associated with evidence translation	7. Integrating the evidence 8. Consulting stakeholders

## Data Availability

The data are not available due to privacy restrictions.

## Supplementary Materials

The following are available online at https://www.mdpi.com/article/10.3390/nu13113748/s1, Table S1: semi-structured interview guide.
