# Supplementary material for: Evidence Use in the Development of the Australian Dietary Guidelines: A Qualitative Study
Source: Nutrients. 2021 Oct 23;13(11):3748. doi: 10.3390/nu13113748 (PMC8620517; doi:10.3390/nu13113748)
Supplement: Supplementary file 1 [file nutrients-13-03748-s001.zip › nutrients-1405036-supplementary.pdf]

Table S1. semi-structured interview guide.

Introduction	• Thank the participant for their time<br>• Introduce myself and the project<br>○ PhD focuses on evidence use in dietary guideline development<br>○ This study is about the Australian Dietary Guidelines (ADGs) in particular<br>• Confirm that signed consent form has been received<br>○ Reiterate that participation is voluntary…<br>○ Participation is confidential…<br>○ Interview data will de-identified and stored safely…<br>○ Audio recorded only…<br>• Check for any further questions about the study<br><br>***PRESS RECORD***
Key concepts	• During the interview we'll talk about…<br>○ Evidence use in development of the current ADGs…<br>○ What could be done differently going forward…<br>• Just to provide some context…<br>○ We know that dietary guidelines should be evidence based, and that many different types of evidence can be used…<br>• When we think about the different types of evidence that can be used, we can think about…<br>○ Exposures: nutrients, foods, dietary patterns<br>○ Outcomes: nutritional adequacy, chronic disease risk<br>○ Study designs: RCTs, observational studies<br>• There are also different ways that this evidence can be reviewed e.g. for the current ADGs:<br>○ Systematic reviews – mostly foods/food groups<br>○ Narrative reviews
Evidence use in development of current dietary guidelines	Can you tell me about your role in the evidence review that informed the current ADGs?<br>• Developing review methods?<br>○ Developing research questions?<br>○ Inclusion criteria and search strategy?<br>○ Quality assessment methods?<br>• Conducting the reviews?<br>○ Conducing the searches?<br>○ Screening search results?<br>○ Extracting data from included studies?<br>○ Conducting quality assessments?<br>• Translating evidence into dietary guidelines?<br>○ Food modelling component?
Evidence use in development of future dietary guidelines	In developing the next iteration of the ADGs, how could the evidence review process be improved?<br>• Development of research questions?<br>• Inclusion criteria and search strategy?<br>• Quality assessment methods e.g. NHMRC, GRADE, other?<br>• Stakeholder involvement?<br>○ Consumers?<br>○ Food industry?<br><br>What types of evidence should be used to inform the next iteration of the ADGs?<br>• Nutrient-based vs food-based vs dietary patterns?<br>• RCTs vs observational studies?<br>• Evidence from other types of research?<br>○ Environmental sustainability?

	○ Equity e.g. food access?<br><br>What are the advantages and disadvantages of using particular types of research to inform the next iteration of the ADGs?<br>In developing the next iteration of the ADGs, do you foresee any challenges associated with using particular types of evidence?<br>• Availability of relevant evidence?<br>• Conflicts of interest?<br><br>If so, how could these challenges be addressed?<br><br>Are there any other comments you'd like to make?
Conclusion	• Thank the participant<br>• Outline the next steps<br>○ Summary of results at completion of the study<br>○ Copy of the journal article once it's been published<br>• Provide an opportunity for questions<br>○ You can contact me at any time
